# EFFECTS OF DIFFERENT EXERCISE INTERVENTION MODALITIES ON BALANCE AND PHYSICAL FUNCTION AMONG OLDER ADULTS

**DOI:** 10.1093/geroni/igae098.0363

**Published:** 2024-12-31

**Authors:** Xiaodong Chen, Lingxiao He, Ya Fang

**Affiliations:** Xiamen University, Xiamen, Fujian, China (People’s Republic); Xiamen University, Xiamen, Fujian, China (People’s Republic); Xiamen University, Xiamen, Fujian, China (People’s Republic)

## Abstract

**Abstract Objective:**

To compare the effects of on-site team (OST) exercise and live-streamed home (LSH) exercise model on balance and physical function among community-dwelling older adults. Design, Setting, and Participants: A 24 week, randomized, controlled, assessor blinded trial was conducted from February 25, 2023 to November 30, 2023. Participants were 297 older adults aged 65 years and older from 11 communities across Xiamen, China.

**Interventions:**

Participants were block randomized to OST and LSH groups with multimodal elastic band-based exercise offline and online (n=99), and a control group with no exercise intervention (n=99). Main outcome measures: The primary outcomes were Berg balance scale scores and the short physical performance battery (SPPB). Secondary outcomes were muscle mass, muscle strength (knee extension strength and grip strength), sleep quality, adherence, and adverse events.

**Results:**

Among 297 randomized older adults (mean age, 71.8[SD, 5.5] years; 52.7% female), 280 (93.8%) completed the trial. Both intervention groups showed increase in Berg balance scale scores and SPPB scores relative to control (adjusted mean differences: OST group vs. control, 1.26 [95% CI, 0.74 to 1.79] and 3.76 [95% CI, 2.25 to 5.26]; P< 0.001; LSH group vs. control: 1.00 [95% CI, 0.44 to 1.56] and 3.42 [95% CI, 1.83 to 5.02]; P< 0.001; OST vs. LSH group, 0.261 [95% CI, -0.26 to 0.78] and 0.33 [95% CI, -1.11 to 1.78]; P=0.463 and 0.853). Similar results were seen in terms of muscle strength.

**Conclusion:**

Live-streamed home exercise is an effective approach to improving balance and physical function among older adults.

